# Sharing Clinical Trial Data: A Proposal from the International Committee of Medical Journal Editors

**DOI:** 10.1371/journal.pmed.1001950

**Published:** 2016-01-20

**Authors:** Darren B. Taichman, Joyce Backus, Christopher Baethge, Howard Bauchner, Peter W. de Leeuw, Jeffrey M. Drazen, John Fletcher, Frank A. Frizelle, Trish Groves, Abraham Haileamlak, Astrid James, Christine Laine, Larry Peiperl, Anja Pinborg, Peush Sahni, Sinan Wu

**Affiliations:** 1 Secretary, ICMJE, Executive Deputy Editor, *Annals of Internal Medicine*; 2 Representative and Associate Director for Library Operations, National Library of Medicine; 3 Chief Scientific Editor, *Deutsches Ärzteblatt (German Medical Journal)*; 4 Editor-in-Chief, *JAMA (Journal of the American Medical Association)* and the JAMA Network; 5 Editor-in-Chief, *Nederlands Tijdschrift voor Geneeskunde (The Dutch Medical Journal)*; 6 Editor-in-Chief, *New England Journal of Medicine*; 7 Editor-in-Chief, *Canadian Medical Association Journal*; 8 Editor-in-Chief, *New Zealand Medical Journal*; 9 Head of Research, *British Medical Journal*; 10 Editor-in-Chief, *Ethiopian Journal of Health Sciences*; 11 Deputy Editor, *The Lancet*; 12 Editor-in-Chief, *Annals of Internal Medicine*; 13 Chief Editor, *PLOS Medicine*; 14 Scientific Editor-in-Chief, *Ugeskrift for Laeger (Danish Medical Journal)*; 15 Representative and Past President, World Association of Medical Editors; 16 Representative, *Chinese Medical Journal*

## Abstract

In a joint publication across 14 member journals, the International Committee of Medical Journal Editors (ICMJE) proposes to require sharing of deidentified individual patient data underlying published clinical trials.

The International Committee of Medical Journal Editors (ICMJE) believes that there is an ethical obligation to responsibly share data generated by interventional clinical trials because participants have put themselves at risk. In a growing consensus, many funders around the world—foundations, government agencies, and industry—now mandate data sharing. Here we outline ICMJE’s proposed requirements to help meet this obligation. We encourage feedback on the proposed requirements. Anyone can provide feedback at www.icmje.org by 18 April 2016.

The ICMJE defines a clinical trial as any research project that prospectively assigns people or a group of people to an intervention, with or without concurrent comparison or control groups, to study the cause-and-effect relationship between a health-related intervention and a health outcome. Further details may be found in the “Recommendations for the Conduct, Reporting, Editing and Publication of Scholarly Work in Medical Journals” at www.icmje.org.

As a condition of consideration for publication of a clinical trial report in our member journals, the ICMJE proposes to require authors to share with others the deidentified individual-patient data (IPD) underlying the results presented in the article (including tables, figures, and appendices or supplementary material) no later than 6 months after publication. The data underlying the results are defined as the IPD required to reproduce the article’s findings, including necessary metadata. This requirement will go into effect for clinical trials that begin to enroll participants beginning 1 year after the ICMJE adopts its data-sharing requirements.^†^

Enabling responsible data sharing is a major endeavor that will affect the fabric of how clinical trials are planned and conducted and how their data are used. By changing the requirements of the manuscripts we will consider for publication in our journals, editors can help foster this endeavor. As editors, our direct influence is logically, and practically, limited to those data underpinning the results and analyses we publish in our journals.

The ICMJE also proposes to require that authors include a plan for data sharing as a component of clinical trial registration. This plan must include where the researchers will house the data and, if not in a public repository, the mechanism by which they will provide others access to the data, as well as other data-sharing plan elements outlined in the 2015 Institute of Medicine Report (e.g., whether data will be freely available to anyone upon request or only after application to and approval by a learned intermediary, whether a data use agreement will be required) [1]. ClinicalTrials.gov has added an element to its registration platform to collect data-sharing plans. We encourage other trial registries to similarly incorporate mechanisms for the registration of data-sharing plans. Trialists who want to publish in ICMJE member journals (or nonmember journals that choose to follow these recommendations) should choose a registry that includes a data-sharing plan element as a specified registry item or allows for its entry as a free-text statement in a miscellaneous registry field. As a condition of consideration for publication in our member journals, authors will be required to include a description of the data-sharing plan in the submitted manuscript. Authors may choose to share the deidentified IPD underlying the results presented in the article under less restrictive, but not more restrictive, conditions than were indicated in the registered data-sharing plan.

ICMJE already requires the prospective registration of all clinical trials prior to enrollment of the first participant. This requirement aims, in part, to prevent selective publication and selective reporting of research outcomes, and to prevent unnecessary duplication of research effort. Including a commitment to a data-sharing plan is a logical addition to trial registration that will further each of these goals. Prospective trial registration currently includes documenting the planned primary and major secondary end points to be assessed, which enables identification of incomplete reporting as well as post hoc analyses. Declaring the plan for sharing data prior to their collection will further enhance transparency in the conduct and reporting of clinical trials by exposing when data availability following trial completion differs from prior commitments.

Sharing clinical trial data, including deidentified IPD, requires planning to ensure appropriate ethics committee or institutional review board approval and the informed consent of study participants. Accordingly, we will defer these requirements for 1 year to allow investigators, trial sponsors, and regulatory bodies time to plan for their implementation.

Just as the confidentiality of trial participants must be protected (through the deidentification of IPD), and the needs of those reasonably requesting data met (through the provision of useable data), the reasonable rights of investigators and trial sponsors must also be protected. ICMJE proposes the following to safeguard these rights. First, ICMJE editors will not consider the deposition of data in a registry to constitute prior publication. Second, authors of secondary analyses using these shared data must attest that their use was in accordance with the terms (if any) agreed to upon their receipt. Third, they must reference the source of the data using a unique identifier of a clinical trial’s data set to provide appropriate credit to those who generated it and allow searching for the studies it has supported. Fourth, authors of secondary analyses must explain completely how theirs differ from previous analyses. In addition, those who generate and then share clinical trial data sets deserve substantial credit for their efforts. Those using data collected by others should seek collaboration with those who collected the data. However, because collaboration will not always be possible, practical, or desired, an alternative means of providing appropriate credit needs to be developed and recognized in the academic community. We welcome ideas about how to provide such credit.

Data sharing is a shared responsibility. Editors of individual journals can help foster data sharing by changing the requirements of the manuscripts they will consider for publication in their journals. Funders and sponsors of clinical trials are in a position to support and ensure adherence to IPD sharing obligations. If journal editors become aware that IPD sharing obligations are not being met, they may choose to request additional information; to publish an expression of concern; to notify the sponsors, funders, or institutions; or in certain cases, to retract the publication.

In the rare situation in which compliance with these requirements is impossible, editors may consider authors’ requests for exceptions. If an exception is made, the reason(s) must be explained in the publication.

Sharing data will increase confidence and trust in the conclusions drawn from clinical trials. It will enable the independent confirmation of results, an essential tenet of the scientific process. It will foster the development and testing of new hypotheses. Done well, sharing clinical trial data should also make progress more efficient by making the most of what may be learned from each trial and by avoiding unwarranted repetition. It will help to fulfill our moral obligation to study participants, and we believe it will benefit patients, investigators, sponsors, and society.

*Feedback may be posted at www.icmje.org by 18 April 2016*.
